# Spatially Uniform ReliefF (SURF) for computationally-efficient filtering of gene-gene interactions

**DOI:** 10.1186/1756-0381-2-5

**Published:** 2009-09-22

**Authors:** Casey S Greene, Nadia M Penrod, Jeff Kiralis, Jason H Moore

**Affiliations:** 1 Department of Genetics, Norris Cotton Cancer Center, Dartmouth Medical School, Lebanon, NH, USA; 2 Department of Community and Family Medicine, Dartmouth Medical School, Lebanon, NH, USA; 3 Department of Computer Science, University of New Hampshire, Lebanon, NH, USA; 4 Department of Computer Science, University of Vermont, Burlington, VT, USA; 5 Translational Genomics Research Institute, Phoenix, AZ, USA

## Abstract

**Background:**

Genome-wide association studies are becoming the de facto standard in the genetic analysis of common human diseases. Given the complexity and robustness of biological networks such diseases are unlikely to be the result of single points of failure but instead likely arise from the joint failure of two or more interacting components. The hope in genome-wide screens is that these points of failure can be linked to single nucleotide polymorphisms (SNPs) which confer disease susceptibility. Detecting interacting variants that lead to disease in the absence of single-gene effects is difficult however, and methods to exhaustively analyze sets of these variants for interactions are combinatorial in nature thus making them computationally infeasible. Efficient algorithms which can detect interacting SNPs are needed. ReliefF is one such promising algorithm, although it has low success rate for noisy datasets when the interaction effect is small. ReliefF has been paired with an iterative approach, Tuned ReliefF (TuRF), which improves the estimation of weights in noisy data but does not fundamentally change the underlying ReliefF algorithm. To improve the sensitivity of studies using these methods to detect small effects we introduce Spatially Uniform ReliefF (SURF).

**Results:**

SURF's ability to detect interactions in this domain is significantly greater than that of ReliefF. Similarly SURF, in combination with the TuRF strategy significantly outperforms TuRF alone for SNP selection under an epistasis model. It is important to note that this success rate increase does not require an increase in algorithmic complexity and allows for increased success rate, even with the removal of a nuisance parameter from the algorithm.

**Conclusion:**

Researchers performing genetic association studies and aiming to discover gene-gene interactions associated with increased disease susceptibility should use SURF in place of ReliefF. For instance, SURF should be used instead of ReliefF to filter a dataset before an exhaustive MDR analysis. This change increases the ability of a study to detect gene-gene interactions. The SURF algorithm is implemented in the open source Multifactor Dimensionality Reduction (MDR) software package available from .

## Background

Technological advances are rapidly improving geneticists ability to measure variation between individuals. Because of these advances, the genome-wide association study is now a common approach to detecting genetic factors which influence individual susceptibility to common human diseases. Genome-wide association studies targeting common variants which, alone, influence susceptibility have produced mixed results [1-5]. As currently performed, these studies ignore complex interactions between variants that may lead to disease susceptibility. These are often ignored because methods to detect these interactions are computationally infeasible or provide insufficient sensitivity.

Epistasis is a term literally meaning "resting upon" which refers to the situation where interacting genes, as opposed to a single gene, influence a trait. Because of the complex architecture of biological networks, epistasis is likely to be fundamental to an individual's disease risk for common human diseases [6]. This, combined with the knowledge that single-locus results have not frequently replicated for common human diseases [7,8], indicates that methods to detect and characterize epistasis are likely to be critical to understanding the genetic basis of common human disease.

Detecting and characterizing epistatic interactions in datasets containing large numbers of SNPs is challenging. It requires examining the effect of SNPs not just in isolation, but also in concert with other SNPs. In a dataset with one million SNPs, a number typically provided by high throughput technologies, there are about 5 × 10^11 ^pairwise combinations of SNPs. For three-way combinations, the number is 1.7 × 10^17^. For higher order interactions the number of combinations is astronomical. Combinatorial methods which evaluate each such combination are not feasible [9].

Efficient algorithms for identifying sets of SNPs likely to contain predictive models for disease susceptibility are therefore needed. Methods of filtering SNPs are one possibility. These first rank the attributes by some criterion. Then either the top *K *SNPs or all SNPs above some threshold *T *are selected. The SNPs within this set can then be analyzed for interactions using combinatorial methods. Stochastic search wrappers are another possibility. These wrappers are probabilistic methods which retain the ability to consider all attributes and have the potential to use information learned early in the search to direct future exploration. Relief algorithms are nearest neighbor based approaches to detecting attributes relevant for some outcome. Relief algorithms are attractive for use in genetic association studies using either filters or wrappers because the computation time required increases linearly with the number of SNPs and quadratically with the number of individuals. Importantly, these algorithms are able to detect attributes associated with disease through interactions or independent main effects, although they do not provide a model for the effect [10]. Instead, information gleaned from these methods can be used as input into other approaches. Stochastic search approaches such as genetic programming [11-13] and ant colony optimization [14] can successfully develop models in this domain when information from the Relief family of algorithms is used to assist the search, although they fail to detect purely epistatic associations without this additional information [12]. Motsinger et al. [15] have shown that patterns of correlation between SNPs can make the problem easier to solve in the absence of expert knowledge, although here we specifically examine uncorrelated SNPs. Moore et al. briefly discuss both filter and wrapper options as part of an overall epistasis analysis strategy for human disease susceptibility [16] and Greene et al. [17] provide a theoretical analysis of both approaches. For the situation where there is a single source of expert knowledge, the filter approach is most appropriate [17]. In this situation we are considering the success rate of individual Relief methods, each of which is a single source which meets these assumptions up to a good approximation according to the appendix (Additional file 1). For this reason we test the ability of these methods to successfully filter a dataset retaining SNPs with an epistatic interaction associated with disease susceptibility.

Numerous variants of Relief have been developed. When applied to genetic association study data these methods use genetically similar individuals or, equivalently, nearest neighbors to adjust weights which are assigned to each SNP. The nearest neighbor is the nearest individual in the dataset to the current individual calculated across all SNPs. While Relief uses, for each individual, a single nearest neighbor in each class, ReliefF, a variant of Relief, uses multiple nearest neighbors, and thus is more robust when the dataset contains noise [18]. Moore and White developed a Tuned ReliefF (TuRF) approach for human genetics [19]. This approach, though requiring more computer time, further improves the performance when the data contain a large number of non-relevant SNPs in addition to a small number of relevant SNPs. TuRF achieves this by iterating a ReliefF algorithm and, with each iteration, deleting SNPs with the lowest ReliefF weights, i.e. those thought to be least predictive [19]. SNPs are assigned a weight based on their normalized weights when removed. This iterative approach improves the overall ranking of disease associated SNPs because noisy SNPs are most often removed. This means that the re-estimation can more accurately evaluate the relevance of the remaining SNPs.

Here we present a new version of Relief, called Spatially Uniform ReliefF or, briefly, SURF. It detects epistatic interactions with a significantly higher success rate than the Relief variant widely used for machine learning, ReliefF. Iterated SURF, called SURF & TuRF, has a significantly higher success rate than TuRF. For each individual SURF, like ReliefF, adjusts weights of all the SNPs by using certain neighbors of the individual. While ReliefF uses a fixed number of nearest neighbors, SURF uses all neighbors within a fixed distance of the individual. This distance may be thought of as a similarity threshold. Thus SURF uses precisely those neighbors more similar than this threshold. ReliefF, on the other hand, may use either fewer or more neighbors, thereby possibly neglecting informative individuals or including uninformative ones. Furthermore, similarity thresholds which give greater success rate than ReliefF can be estimated from the data while distances are pre-computed, thus removing a nuisance parameter from the algorithm (see §2 in the appendix). SURF also does not increase the complexity of the algorithm, so the scaling is still linear with respect to the number of SNPs and quadratic with respect to the number of individuals.

### Relief and Spatially Uniform ReliefF (SURF)

All Relief algorithms attach a weight to each SNP. The higher the weight of a SNP, the more likely it is predictive of disease status. Genetically similar individuals are used to adjust these SNP weights. We define the distance between two individuals as the number of their SNPs with differing genotypes. With this distance metric, nearest neighbors share genotypes at the greatest number of SNPs, and so are genetically most similar.

**Figure 1:**
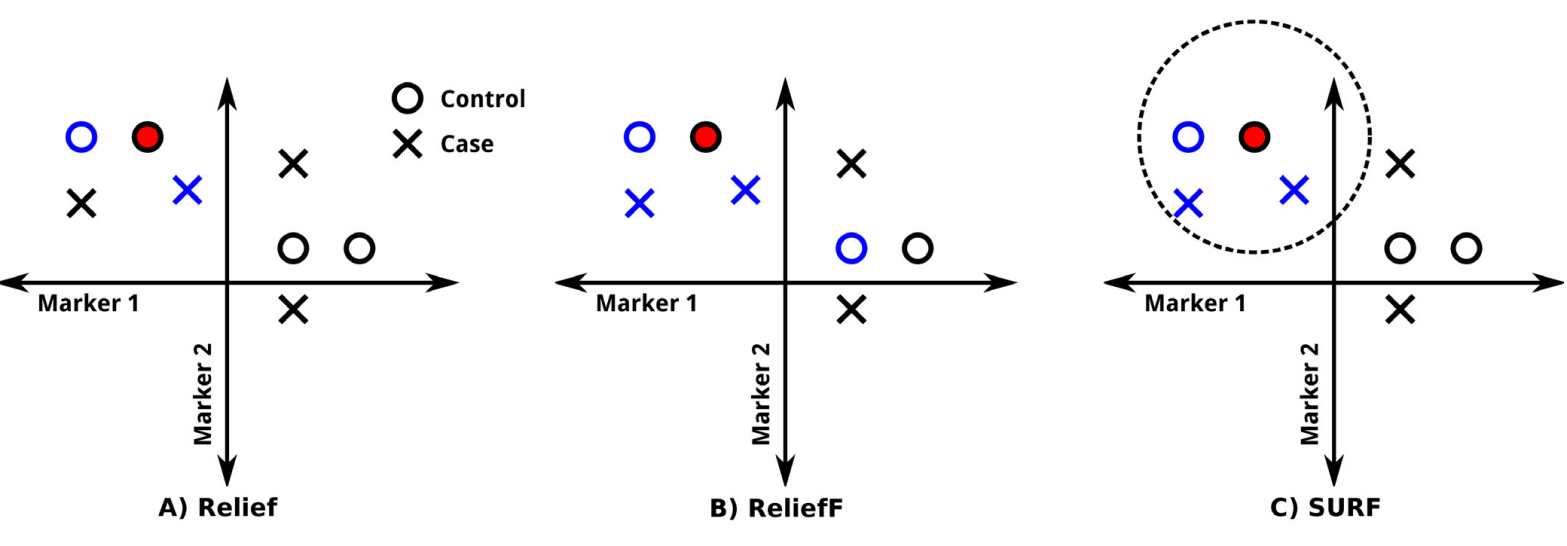
**How Relief, ReliefF and SURF select neighbors**. Each panel in this figure shows the genotypes at two markers for a dataset of cases and controls. For the purpose of this example only these two markers will be considered and both are continuous. When analyzing real data, the process of selecting neighbors is the same, however, but there will be thousands of discrete valued markers (SNPs) each of which would be represented by one of thousands of dimensions. The individual for whom neighbors are being found is shown by the filled red circle. The neighbors that each approach uses for weighting are highlighted in blue. Parts A, B, and C represent how Relief, ReliefF and SURF would select neighbors to be used in weighting. Relief selects the nearest individual of the same class (blue circle) and the nearest individual of the other class (blue cross). ReliefF selects some user specified number of individuals (two in this example) to be used for weighting. SURF, instead of using a fixed number of neighbors, uses all individuals within a distance threshold. The dotted line shows a hypothetical distance threshold.

Relief algorithms are based on the assumption that those SNPs of nearby individuals which have different states (i.e. differing genotypes) are either most or least predictive of disease status. Relief algorithms adjust the weights of these SNPs-upward if the two individuals have different disease status, and downward by the same amount if they have the same status. More precisely, the original Relief algorithm adjusts, for each individual *I*_*i*_, the SNP weights using *I*_*i*_'s nearest hit (the individual which is closest to *I*_*i *_and in the same class as *I*_*i*_) and *I*_*i*_'s nearest miss (the individual which is closest to *I*_*i *_and in the other class from *I*_*i*_). In the case of SURF, for each individual *I*_*i*_, this adjustment is done using each hit and miss within a fixed threshold distance *T *of *I*_*i*_. Figure 1 shows graphically how neighbors are selected with each Relief algorithm.

Relief is able to detect epistatic SNPs, even when no single SNP has an effect. We outline how it does this for epistatic pairs. More detail is in the appendix (Additional file 1). All of the penetrance functions used in this work are available in Additional file 2. We begin with a discussion of epistatic pairs. Consider the penetrance function for the epistatic pair of SNPs shown in Table 1. If an individual has genotype AA and the genotype of SNP_2 _is unknown, then the probability the individual is sick is

**Table 1:** Penetrance values for an example epistasis model with a heritability of 0.1.

		***SNP***_1_		
		
		**AA (0.36)**	**Aa (0.48)**	**aa (0.16)**
*SNP*_2_	BB (0.36)	0.469	0.198	0.754
	
	Bb (0.48)	0.337	0.502	0.141
	
	bb (0.16)	0.339	0.453	0.285



The individual has the same probability of being sick if he has genotype either Aa or aa, provided again that the genotype of SNP_2 _is unknown. Similarly, if his genotype is either BB, bB or bb with SNP_1_'s genotypes unknown, the probability he is sick is again .3849. The point is that no single SNP has an effect on disease susceptibility. Only the relevant pair does.

Now we discuss how Relief detects epistatic pairs. Given an individual *I*_*i*_, we define the set *M*_*k*Δ _to consist of those misses with exactly *k *of their two relevant SNPs in a different state from those of individual *I*_*i*_. In the case of two relevant SNPs, *k *= 0, 1 or 2. Note that the miss nearest *I*_*i *_is in exactly one of the three sets *M*_0Δ_, *M*_1Δ _or *M*_2Δ_. Indeed, these partition the set of all misses. The sizes of the sets *M*_*k*Δ _can be determined (as in §1 of the appendix) from the penetrance function which governs the relationship between genotype and phenotype. As an example, with a sample size of 1600 and the penetrance function shown in Table 1 the sizes of these sets are

(1)

For the analogous sets involving hits we have

(2)

These are actually expected numbers rounded to the nearest integer. Since |*M*_1Δ_| > |*M*_2Δ_|, the contribution of the irrelevant SNPs to the distance from *I*_*i *_to its nearest point in *M*_1Δ _tends to be less than that to its nearest point in *M*_2Δ_. The two relevant SNPs contribute one to the distance from *I*_*i *_to every point of *M*_1Δ_. For points in *M*_2Δ_, the contribution to this distance is two, which makes points in *M*_2Δ _farther by one from *I*_*i*_, on average, than points in *M*_1Δ_. Since the states of the relevant and irrelevant SNPs are independent, it follows that the nearest miss is more likely to be in *M*_1Δ _than *M*_2Δ_. To be precise for the example in table 1 the probability, according to equation (10) of the appendix, that the closest miss is in *M*_1Δ _is



while the probability it is in *M*_2Δ _is



We mention that the probability it is in *M*_0Δ _is



but do not use this since Relief adjusts weights only for SNPs where pairs of individuals have differing genotypes. The analogous probabilities for hits are



If the nearest miss is in *M*_2Δ_, then the Relief score of both relevant SNPs is increased by one. If it is in *M*_1Δ_, there is a 50% chance that the score of the first relevant SNP is increased by one. Thus the expected contribution due to misses of individual *I*_*i *_to the score of a relevant SNP is



Using the same notation for hits, except with H in place of M, an analogous discussion gives



as the expected contribution dues to hits of individual *I*_*i *_to the score of a relevant SNP. Thus the expected contribution of individual *I*_*i *_to the score of a relevant SNP is

(3)

The value of this for the example we have been considering is .005. The expected contribution of individual *I*_*i *_to the score of an irrelevant SNP is 0. This indicates why Relief tends to assign higher scores to relevant SNPs than to irrelevant ones.

The analysis of SURF, though mathematically easier, is more subtle. Again, let *I*_*i *_be a random, but fixed, individual. Then, as before, each miss within the threshold distance *T *of *I*_*i *_is in one of the three sets *M*_0Δ_, *M*_1Δ _or *M*_2Δ_. For *k *= 0, 1 and 2, let *TM*_*k *_be the subset of *M*_*k*Δ _consisting of those individuals within distance *T *of *I*_*i*_. Using analogous notation for hits with *H *in place of *M*, the mean contribution of individual *I*_*i *_to the SURF score of a relevant SNP is

(4)

The  is here since each individual in *TM*_1 _and *TH*_1 _changes the score of a relevant SNP by , on the average.

Returning now to the example model, specifically expressions (1) and (2), we see that |*M*_2Δ_| - |*H*_2Δ_| < 0.

Thus, on average, |*TM*_2_| - |*TH*_2_| < 0; however two factors make  > 0. Namely



making



Also, elements of *M*_1Δ _and *H*_1Δ _are, on average, one closer to *I*_*i *_than elements of *M*_2Δ _and *H*_2Δ_. Together these make



and, consequently,  > 0, on average. For the example penetrance function, equation (3) of the appendix gives  = .519; however, this SURF score cannot be reasonably compared to the analogous Relief score of .005 without a discussion of the variances of these scores. We do this in the appendix, and also indicate in §5 why SURF outperforms ReliefF using 10 nearest neighbors.

The scores  depend on the value of the distance threshold *T*. In our simulations, we have chosen *T *to maximize the . The final score of a relevant SNP is the sum of the  values for each individual.

Because of the way the variance of this sum varies with *T*, slightly smaller values of *T *are probably optimal. This is discussed at the end of §2 of the appendix.

## Results and Discussion

**Figure 2:**
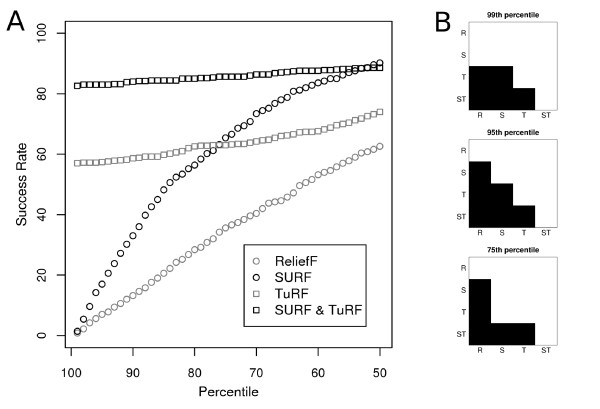
**Example Success Rate and Significance of Differences**. Part A shows the detailed success rate analysis results for a single heritability (0.1) and sample size (1600). The success rate to filter both relevant SNPs into percentiles from the 99^*th *^to 50^*th *^is shown. The 99^* th*^percentile corresponds to the top 10 SNPs by the assigned weights in these datasets which contain 1000 SNPs. In part B pairwise comparisons are made between each pair of methods at the 99^*th*^, 95^*th*^, and 75^*th *^percentiles. ReliefF, SURF, TuRF, and SURF&TuRF are labeled R, S, T, and ST respectively. Significance is illustrated with levels of grey (i.e. light grey indicates 0.01 <*p *≤ 0.05, dark grey indicates 0.001 <*p *≤ 0.01, and black indicates *p *≤ 0.001). As an example, at the 99^*th *^percentile the blank square at the intersection of R and S indicates that the difference between ReliefF and SURF was not significant. On the other hand the black square at the intersection of S and ST indicates that the difference between the success rates of SURF and SURF&TuRF at that percentile was highly significant.

Our results suggest that the SURF approaches provide a more successful method for the detection of gene-gene interactions in these data. Figure 2 shows both success rate and significance test results for a single sample size and heritability (1600 and 0.1 respectively). These results indicate that the success rates of the SURF approaches (SURF and SURF & TuRF) are greater than their corresponding ReliefF approaches (ReliefF and TuRF). Furthermore the step plots show that this difference is highly significant except for the 99^*th *^percentile comparison of ReliefF and SURF. Neither of the non-iterative approaches is highly effective for filtering to the 99^*th *^percentile for this heritability and sample size, so as a stringent filter the iterative approaches are most useful.

**Figure 3:**
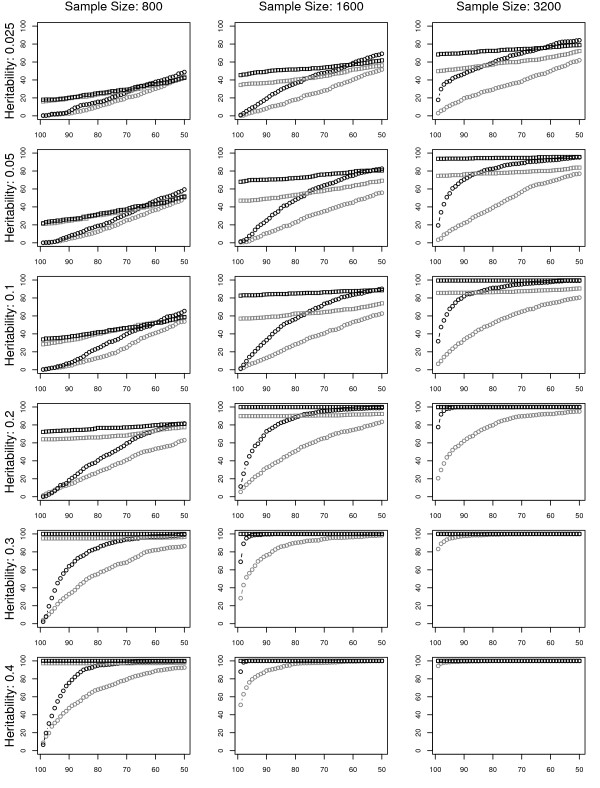
**Success Rate Analysis**. This is a summary of success rate as shown in figure 2 across a wide range of sample sizes and heritabilities. Within each heritability the success rates for all five genetic models for that heritability are averaged. The *x*-axis for each plot corresponds to the percentiles as in figure 2. Across these situations, SuRF alone performs as well as TuRF when filtering to the 75^*th *^percentile of SNPs. SURF outperforms ReliefF, the tuned approaches outperform the non-tuned approaches when using a more stringent filter (i.e. 99^*th *^and 95^*th *^percentiles), and SURF & TuRF outperforms TuRF with ReliefF.

Our complete results, shown in figure 3, show that the new SURF algorithm, outperforms ReliefF. Furthermore we see that this increase in success rate is not redundant with the tuned approaches, as both of these, TuRF and SURF & TuRF, which iteratively remove attributes with low quality estimates, are much better than the standard Relief and SURF approaches at selecting a small subset which contains the functional attributes. Here we see that these approaches significantly outperform ReliefF and SURF when the task is to filter the dataset to the 99^*th *^or 95^*th *^percentiles of SNPs. Finally we find that SURF & TuRF outperforms TuRF alone achieving a much greater success rate, particularly at moderate heritabilities. We find that these differences are statistically significant. The success rate when SURF is used, particularly with larger sample sizes, is consistently significantly greater than the success rate when the standard method, ReliefF is used (see Additional files 3, 4, 5) for both the "tuned" and non-iterative approaches. Additionally the success rates of these "tuned" algorithms to include the proper SNP in the 99^*th *^and 95^*th *^percentiles are consistently significantly better than the success rates of the non-tuned approaches (see figure 2 and Additional files 3, 4, 5).

Methods which increase success rate without an increase in computational complexity or sample size are extremely desirable for genome-wide association studies. By developing improved methods for detecting epistasis we greatly expand our ability to characterize interactions in large datasets. Moore argues that when people use sensitive methods to detect epistasis, they are frequently able to find examples of it [20]. Algorithms which both detect and characterize epistasis in the absence of main effects are of combinatorial complexity for the number of SNPs. The SURF algorithm we introduce to detect disease associated interacting SNPs is, like ReliefF, of linear complexity for the number of SNPs. Moreover, its success rate for epistasis analysis is higher than ReliefF's. One caveat is that Relief methods such as SURF, though useful for detecting interacting SNPs, neither identify specific interacting pairs nor develop a model. Because SURF & TuRF is able to detect interacting genetic variants which are predictive of human health, weights from this algorithm can be used to filter a dataset before traditional combinatorial approaches are used to characterize the interaction. McKinney et al. have previously integrated ReliefF [21] and TuRF [22] with other information sources using an evaporative cooling technique to direct genetic association analyses. Direct replacement of ReliefF by SURF & TuRF may improve the sensitivity of these frameworks to detect and characterize interactions.

SURF & TuRF's greatly increased success rate to detect epistasis improves our ability to detect variants leading to disease risk in the absence of main effects. This new distance based approach may also be extensible to biological and biomedical data beyond case-control genetic association studies. While ReliefF, which SURF & TuRF builds on, is usable for these discrete endpoints and attribute values, other modifications to ReliefF have extended this machine learning method to other data types. With Regression ReliefF (RReliefF), ReliefF is broadened to handle continuous attributes and endpoints [23,24]. Future work should examine whether the new distance based approach used for SURF & TuRF also improves these methods. If using a distance threshold also improves RReliefF methods, the sensitive SURF approach can be applied to continuous gene expression data or to detecting variants predictive of continuous endpoints. With future work it may also be possible to combine continuous and discrete attributes, to provide a method capable of examining gene-gene, gene-environment, and environment-environment interactions in a common framework.

## Conclusion

Now that it is technically and economically feasible to measure large numbers of DNA sequence variations in human genetics, the bioinformatics challenge is to identify and improve methods for detecting variants which are predictive of disease risk. This is particularly challenging when the task is to identify polymorphisms which have little or no independent effect. The Relief family of algorithms provides one potential solution for SNP selection, and SURF & TuRF is a novel within this family which effectively detects epistasis. By developing sensitive and computationally efficient methods capable of detecting epistasis, it becomes more practical to probe datasets for these interactions. Highly sensitive methods will allow researchers to better understand the impact of epistasis on human health. Both SURF and SURF & TuRF have been included as filtering methods in the user friendly open source Multifactor Dimensionality Reduction (MDR) software package.

## Methods

As discussed SURF weights can be used for genetic analysis in either filters or probabilistic wrappers. Here we consider the simpler filter approach. Specifically we analyze SURF's ability to filter a dataset to the 99^*th*^, 95^*th *^and 75^*th *^percentiles of SNPs without removing those SNPs with an interaction effect predictive of disease susceptibility. ReliefF has previously been used in the genetic analysis of complex diseases in this fashion [25].

The goal of our simulation study is to generate artificial datasets with high concept difficulty to evaluate SURF in the domain of human genetics. We first develop 30 different penetrance functions (i.e. genetic models) which determine the relationship between genotype and phenotype in our simulated data. These functions determine the probability that an individual has disease given his or her genotype. This probability depends only on the genotypes of the two interacting SNPs, not on the genotype of any one SNP. The 30 penetrance functions include groups of five with heritabilities of 0.025, 0.05, 0.1, 0.2, 0.3, or 0.4. These heritabilities range from very small to large genetic effect sizes. Each functional SNP has two alleles with frequencies of 0.4 and 0.6. These models are included in Additional file 2. Each of the models is used to generate 100 replicate datasets with sample sizes of 800, 1600, and 3200. Each dataset consists of an equal number of case (diseased) and control (disease free) subjects. Each pair of functional SNPs is added to a set of 998 irrelevant SNPs for a total of 1000 attributes. A total of 9,000 datasets are generated and analyzed.

We test each method with the following parameters. All four methods can use some or all of the dataset when performing weight estimations. Here we use the entire dataset, as this is similar to what is performed in practice where the number of individuals is often more limiting than the computational costs. ReliefF and TuRF require a number of neighbors. Here we use 10, as suggested by Robnik-Sikonja and Kononenko [24] in a comprehensive analysis. SURF requires a distance threshold. Our theoretical analysis in §2 of the appendix (Additional file 1) suggests that the mean distance between all pairs of individuals in the dataset and across all attributes can be used and thus we use this distance in this situation. By using the mean distance as calculated from the data, we remove this nuisance parameter from the algorithm. Both SURF & TuRF and TURF remove a number of SNPs at each iteration before re-estimating the weights of the remaining SNPs. Here we remove 25 SNPs at each iteration (2.5% of the dataset).

Here, because we are interested in interactions, we consider the success rate to be the number of times that both relevant SNPs are scored above a given threshold. We set this stricter standard here because further analysis steps can not succeed of both relevant parts of the interaction are not discovered. To estimate the success rate, we use 100 datasets for each of the 30 models. Specifically, the percentage of datasets for which a method ranks the two relevant SNPs above the *N*^*th *^percentile of all SNPs is the estimate of the method's success rate. We apply Fisher's exact test to assess the significance of differences between the success rates of the tested methods at these thresholds. These percentiles represent the situation where each method is used to filter a large dataset with 1000 SNPs to a smaller dataset of 10, 50, and 250 SNPs respectively. Fisher's exact test is a significance test appropriate for categorical count data [26]. The resulting *p*-value for this test can be interpreted as the likelihood of seeing a difference of the size observed among success rates when the methods do not differ. We consider results statistically significant when *p *≤ 0.05. Additionally, we graphically show results for filtering to each percentile from the 99^*th *^to the 50^*th*^.

## Competing interests

The authors declare that they have no competing interests.

## Authors' contributions

CSG and JK developed SURF. CSG, NMP and JHM designed and performed the experiments. CSG, NMP, JK and JHM prepared the manuscript. All authors have read and approved the final manuscript.

## Supplementary Material

Additional file 1 **Appendix**. This is an appendix to accompany the manuscript that includes additional theoretical analysis of the Relief algorithms discussed in the manuscript. Click here for file

Additional file 2 **Epistasis models**. These are the epistasis models used in our data simulation. Click here for file

Additional file 3 **Significance of differences with a sample size of 800**. This is a plot showing the significance of statistical results for the situation where there are 400 cases and 400 control individuals. These plots follow the example shown in Figure 2. Pairwise comparisons are made between each pair of methods at the 99^*th*^, 95^*th*^, and 75^*th *^percentiles. ReliefF, SURF, TuRF, and SURF & TuRF are labeled R, S, T, and ST respectively. Significance is illustrated with levels of grey (i.e. light grey indicates 0.01 <*p *≤ 0.05, dark grey indicates 0.001 <*p *≤ 0.01, and black indicates *p *≤ 0.001). Click here for file

Additional file 4 **Significance of differences with a sample size of 1600**. This is a plot showing the significance of statistical results for the situation where there are 800 cases and 800 control individuals. These plots follow the example shown in Figure 2. Pairwise comparisons are made between each pair of methods at the 99^*th*^, 95^*th*^, and 75^*th *^percentiles. ReliefF, SURF, TuRF, and SURF&TuRF are labeled R, S, T, and ST respectively. Significance is illustrated with levels of grey (i.e. light grey indicates 0.01 <*p *≤ 0.05, dark grey indicates 0.001 <*p *≤ 0.01, and black indicates *p *≤ 0.001). Click here for file

Additional file 5 **Significance of differences with a sample size of 3200**. This is a plot showing the significance of statistical results for the situation where there are 1600 cases and 1600 control individuals. These plots follow the example shown in Figure 2. Pairwise comparisons are made between each pair of methods at the 99^*th*^, 95^*th*^, and 75^*th *^percentiles. ReliefF, SURF, TuRF, and SURF&TuRF are labeled R, S, T, and ST respectively. Significance is illustrated with levels of grey (i.e. light grey indicates 0.01 <*p *≤ 0.05, dark grey indicates 0.001 <*p *≤ 0.01, and black indicates *p *≤ 0.001). Click here for file
