# Supplementary material for: Spatially Uniform ReliefF (SURF) for computationally-efficient filtering of gene-gene interactions
Source: BioData Min. 2009 Sep 22;2:5. doi: 10.1186/1756-0381-2-5 (PMC2761303; doi:10.1186/1756-0381-2-5)

Appendix

In this appendix we use probability theory to analyze SURF and ReliefF. This enables us to approximate the success rates of these methods for any sample size and any epistatic two-way penetrance function. We first discuss penetrance functions, then SURF and ReliefF, and conclude with examples involving two specific penetrance functions.

1 Penetrance functions

We assume that we are given a sample of N individuals, each of class either s, for sick, or w, for well. Two relevant, epistatic SNPs, say SNP 1 and SNP 2, govern the distribution of these classes according to a given penetrance function. All other SNPs, numbered 3 through $N_I + 2$, have no effect.

We also assume that each SNP of each individual is in one of three states: 1, 2, or 3 corresponding to the genotypes AA, Aa, and aa, respectively. Let p_{ij} be the probability that SNP i of a random individual is in state j, and put $\vec{P}_i = \langle p_{i1}, p_{i2}, p_{i3}\rangle$, a vector associated with the ith SNP. We assume Hardy–Weinberg equilibrium holds. So if the frequency of the major allele of SNP i is p, then $p_{i1} = p^2$, $p_{i2} = 2p(1-p)$ and $p_{i3} = (1-p)^2$. The states of different SNPs are independent. Thus $P(ij) = p_{1i}p_{2j}$, where $P(ij)$ is the probability an individual has genotype ij, meaning SNP 1 has state i and SNP 2 state j.

Set $f_{ij} = P(s|ij)$, the probability that an individual is in class s if his genotype is ij. These probabilities are given by the penetrance function. Let $\vec{C}_i = \langle f_{1i}, f_{2i}, f_{3i}\rangle$ and $\vec{R}_i = \langle f_{i1}, f_{i2}, f_{i3}\rangle$ for $i = 1$, 2 and 3. (The six vectors $\vec{R}_i$ and $\vec{C}_i$ are just the rows and columns of the penetrance table.) To say that SNP 1 and SNP 2 are an epistatic pair means that all six marginal penetrances $\vec{P}_1 \cdot \vec{C}_i$ and $\vec{P}_2 \cdot \vec{R}_i$, $i = 1$, 2, 3 are equal. Their common value is often denoted by k, and is the probability an individual is sick regardless of her genotype.

We will often use $P(ij|s)$, the probability a sick individual has genotype ij. By Bayes' formula

$$P(ij|s) = \frac{P(ij)P(s|ij)}{k} = \frac{p_{1i}p_{2j}f_{ij}}{k}.$$

Similarly

$$P(ij|w) = \frac{P(ij)P(w|ij)}{1-k} = \frac{p_{1i}p_{2j}(1-f_{ij})}{1-k}.$$

These, together with the assumption that SNPs 1 and 2 are epistatic give, for fixed i,

$$\sum_{j=1}^{3} P(ij|s) = \frac{p_{1i}}{k}\sum_j p_{2j}f_{ij} = \frac{p_{1i}}{k}\vec{P}_2 \cdot \vec{R}_i = p_{1i}$$

and

$$\sum_{j=1}^{3} P(ij|w) = \frac{p_{1i}}{1-k}\sum_j p_{2j}(1-f_{ij}) = \frac{p_{1i}}{1-k}\left(\left(\sum_j p_{2j}\right) - \vec{P}_2 \cdot \vec{R}_i\right) = p_{1i}.$$

Similarly, for fixed j,

$$\sum_{i=1}^{3} P(ij|s) = p_{2j} = \sum_{i=1}^{3} P(ij|w).$$

For $n = 0$, 1 and 2, let $P_H(n\Delta)$ be the probability that exactly n of the two relevant SNPs change state in going from an arbitrary individual to another of the same class. Similarly,

$P_M(n\Delta)$ are, by definition, probabilities of numbers of state changes for arbitrary misses, rather than hits. There are two types of hits: sick-to-sick and well-to-well. Probabilities involving these analogous to the $P_H(n\Delta)$ are denoted by $P_{ss}(n\Delta)$ and $P_{ww}(n\Delta)$. We have

$$P_{ss}(0\Delta) = \sum_{i,j=1}^{3} (P(ij|s))^2 \quad \text{and} \quad P_{ww}(0\Delta) = \sum_{i,j=1}^{3} (P(ij|w))^2 .$$

Also,

$$\begin{aligned} P_{ss}(1\Delta) &= \sum_{i,j=1}^{3} P(ij|s)\Big(\sum_{\ell\neq j} P(i\ell|s) + \sum_{k\neq i} P(kj|s)\Big) \\ &= \sum_{i,j=1}^{3} P(ij|s)\Big(\big(p_{1i} - P(ij|s)\big) + \big(p_{2j} - P(ij|s)\big)\Big) \\ &= ||\vec{P}_1||^2 + ||\vec{P}_2||^2 - 2P_{ss}(0\Delta), \end{aligned}$$

where $||\vec{P}_i||$ is the norm of the vector $\vec{P}_i$. For the last equality we have used that $\sum_{i,j} P(ij|s) = 1$ and

$$\sum_{i,j=1}^{3} P(ij|s)\, p_{1i} = \sum_j P(1j|s)\, p_{11} + \sum_j P(2j|s)\, p_{12} + \sum_j P(3j|s)\, p_{13} = ||\vec{P}_1||^2.$$

A similar computation shows that

$$P_{ww}(1\Delta) = ||\vec{P}_1||^2 + ||\vec{P}_2||^2 - 2P_{ww}(0\Delta).$$

Assuming that the number of sick individuals in the sample equals the number of well ones, we have

$$P_H(0\Delta) = \frac{1}{2}\big(P_{ss}(0\Delta) + P_{ww}(0\Delta)\big)$$

and

$$P_H(1\Delta) = \frac{1}{2}\big(P_{ss}(1\Delta) + P_{ww}(1\Delta)\big) = ||\vec{P}_1||^2 + ||\vec{P}_2||^2 - 2P_H(0\Delta).$$

Similar computations for misses give

$$P_M(0\Delta) = \sum_{i,j} P(ij|s)P(ij|w) \quad \text{and} \quad P_M(1\Delta) = ||\vec{P}_1||^2 + ||\vec{P}_2||^2 - 2P_M(0\Delta).$$

Computing $P_M(2\Delta)$ directly or using the fact that $\sum_{n=0}^{2} P_M(n\Delta) = 1$ gives

$$P_M(2\Delta) = P_M(0\Delta) + 1 - (||\vec{P}_1||^2 + ||\vec{P}_2||^2).$$

Likewise,

$$P_H(2\Delta) = P_H(0\Delta) + 1 - (||\vec{P}_1||^2 + ||\vec{P}_2||^2).$$

It now follows that

$$P_H(2\Delta) - P_M(2\Delta) = \frac{1}{2}\Big(P_M(1\Delta) - P_H(1\Delta)\Big) = P_H(0\Delta) - P_M(0\Delta). \tag{1}$$

We note that the derivation of this requires that SNPs 1 and 2 are epistatic. We will see that the quantity $P_H(0\Delta) - P_M(0\Delta)$ in equation (1) is a measure of how well SURF detects the relevant SNPs. It is a considerably more accurate measure of this than heritability.

2 SURF

Let SNP i be any SNP, relevant or not. The probability that it is in the same state, or matches, in two randomly chosen individuals is $||P_i||^2$. We now assume that all N_I irrelevant SNPs have the same major allele frequency. Then the number of matching irrelevant SNPs in a random pair of individuals satisfies the binomial distribution corresponding to the Bernoulli trials process with N_I trials and probability of success $||\vec{P}||^2$, where $\vec{P} = \vec{P}_3$. By the Central Limit theorem we have, to very good approximation

$$P(\geq d \text{ matching irrelevant SNPs in a random pair}) = p(d) = \frac{1}{\sqrt{2\pi}} \int_{a(d)}^{\infty} e^{-\frac{1}{2}x^2} dx, \qquad (2)$$

where

$$a(d) = \frac{d - \frac{1}{2} - N_I||\vec{P}||^2}{\sqrt{N_I||\vec{P}||^2(1 - ||\vec{P}||^2)}}.$$

Now fix a random individual I_i. This partitions the sample set of individuals (with I_i removed) into six sets $H_{j\Delta}$ and $M_{j\Delta}$, where $j = 0, 1$ and 2. The set $H_{j\Delta}$ consists of all individuals in the same class as I_i with exactly j relevant SNPs in a different state from those of I_i. The $M_{j\Delta}$ are defined similarly with j the number of state changes between misses. The cardinalities of these sets are

$$|M_{j\Delta}| = P_M(j\Delta)\frac{N}{2} \quad \text{and} \quad |H_{j\Delta}| = P_H(j\Delta)\frac{N}{2},$$

where N is the sample size. (These are really expected cardinalities, but we use them as approximations. We also neglect, for now, the fact that there is one fewer hit than miss.)

Let $b(n, p)$ be the random variable giving the number of successes in a Bernoulli trials process consisting of n trials with p the probability of success of each. The random variable $b(|M_{1\Delta}|, p(d-1))$ then counts the number of individuals in the set $M_{1\Delta}$ with $d-1$ or more irrelevant SNPs in the same state as those of the fixed individual I_i. This is the same as the number of individuals in this set having distance $\leq 1000-d$ from I_i. Similarly $b(|M_{2\Delta}|, p(d))$ is the number in the set $M_{2\Delta}$ with d or more matching SNPs, and $b(|M_{0\Delta}|, p(d-2))$ the number in the the set $M_{0\Delta}$ with such SNPs. The random variable

$$S_i^M = b(\frac{|M_{1\Delta}|}{2}, p(d-1)) + b(|M_{2\Delta}|, p(d))$$

then approximates part of individual I_i's contribution to the relief score of a relevant SNP. This part comes from all misses within distance $1000 - d$ of I_i. The random variable

$$S_i^H = b(\frac{1}{2}|H_{1\Delta}|, p(d-1)) + b(|H_{2\Delta}|, p(d))$$

gives the corresponding contribution from hits. The total contribution of individual I_i to the (unnormalized) relief score of a relevant SNP using those hits and misses with distance $\leq 1000 - d$ from I_i is

$$S_i^R = S_i^M - S_i^H.$$

Up to the approximation given by the Central Limit Theorem, the probability density functions (or, briefly, the PDFs) of the random variables $b(n, p)$ are gaussians. Since convolution preserves these, the probability density function of S_i^R is also a gaussian, so is determined by its mean and variance.

The mean of S_i^R is

$$
\begin{aligned}
M(S_i^R) &= p(d-1)\,\frac{1}{2}\big(|M_{1\Delta}| - |H_{1\Delta}|\big) + p(d)\big(|M_{2\Delta}| - |H_{2\Delta}|\big) \\
&= \frac{N}{2}\Big(p(d-1)\,\frac{1}{2}\big(P_M(1\Delta) - P_H(1\Delta)\big) - p(d)\,\big(P_H(2\Delta) - P_M(2\Delta)\big)\Big) \\
&= \frac{N}{2}\big(p(d-1) - p(d)\big)\big(P_H(0\Delta) - P_M(0\Delta)\big) \qquad \text{using equation (1)} \\
&= \frac{N}{2}\left(\frac{1}{\sqrt{2\pi}}\int_{a(d-1)}^{a(d)} e^{-\frac{1}{2}x^2}dx\right)\big(P_H(0\Delta) - P_M(0\Delta)\big). \qquad (3)
\end{aligned}
$$

Regardless of the penetrance function, this has maximum value when d is chosen so that $0 \in [a(d-1), a(d)]$, or when d is $\frac{1}{2} + N_I||\vec{P}||^2$ rounded up to the nearest integer.

The variance of S_i^R is

$$
\begin{aligned}
V(S_i^R) &= \frac{1}{2}p(d-1)(1-p(d-1))\big(|M_{1\Delta}| + |H_{1\Delta}|\big) + p(d)(1-p(d))\big(|M_{2\Delta}| + |H_{2\Delta}|\big) \\
&\approx \frac{N}{2}p(d-1)(1-p(d-1))\big(\frac{1}{2}P_M(1\Delta) + P_M(2\Delta) + \frac{1}{2}P_H(1\Delta) + P_H(2\Delta)\big) \\
&= N\,p(d-1)(1-p(d-1))\,P(\Delta), \qquad (4)
\end{aligned}
$$

where $P(\Delta) = 1 - ||\vec{P}||^2$ is the probability that an irrelevant SNP has different states in two arbitrary individuals.

Next we consider the score of an arbitrary, irrelevant SNP, say SNP k. We continue to assume that I_i is a random, fixed individual, and $M_{j\Delta}$ and $H_{j\Delta}$ the associated partitioning sets. Let H_Δ be the subset of those individuals in the same class as I_i with the state of SNP k differing from that of I_i. Define a subset M_Δ of misses similarly. Individual I_i's contribution to the score of SNP k is given by the random variable

$$
S_i^I = \sum_{i=0}^{2}\big(b(|M_\Delta \cap M_{i\Delta}|, p_1(d-2+i)) - b(|H_\Delta \cap H_{i\Delta}|, p_1(d-2+i))\big).
$$

Here $p_1(d)$ is defined just as $p(d)$ was in equation (2), except with $N_I - 1$ in place of N_I. Since the states of an irrelevant SNP are independant of those of all other SNPs, we have $|M_\Delta \cap M_{i\Delta}| \approx P(\Delta)P_M(i\Delta)\frac{N}{2}$, and so the mean of S_i^I,

$$
\begin{aligned}
M(S_i^I) &\approx \frac{N}{2}P(\Delta)\sum_{i=0}^{2}(P_M(i\Delta) - P_H(i\Delta))p_1(d-2+i) \\
&= \frac{N}{2}P(\Delta)(P_H(0\Delta) - P_M(0\Delta))\big(2p_1(d-1) - p_1(d-2) - p_1(d)\big).
\end{aligned}
$$

The variance is

$$
\begin{aligned}
V(S_i^I) &= \frac{N}{2}P(\Delta)\sum_{i=0}^{2}(P_H(i\Delta) + P_M(i\Delta))p_1(d-2+i)(1-p_1(d-2+i)) \\
&\approx \frac{N}{2}P(\Delta)\sum_{i=0}^{2}(P_H(i\Delta) + P_M(i\Delta))p(d-1)(1-p(d-1)) \\
&= N\,p(d-1)(1-p(d-1))\,P(\Delta). \qquad (5)
\end{aligned}
$$

Next we work towards finding the values of d which makes SURF most effective, that is most likely to assign higher scores to relevant SNPs than to irrelevant ones. The functions

$M(S_i^R)$, $V(S_i^R)$ and $V(S_i^I)$ of d are all decreasing, or very nearly so, on the interval $[b, N_I+2]$. Here $b \approx \frac{1}{2} + N_I||\vec{P}||^2$ is the value of d which maximizes $M(S_i^R)$ or, equivalently, the one such that $p(d)$ is closest to 1/2. The function $M(S_i^I)$ is ≈ 0 on $[b, N_I+2]$. (It's actually slightly < 0 here, attaining its minimum near $d = b + \sqrt{N_I||\vec{P}||^2(1-||\vec{P}||^2)}$.)

Let $S_{i,1}^R$ and $S_{i,2}^R$ be individual I_i's contributions to the scores of the two relevant SNPs. A computable, but crude measure of the effectiveness of SURF is the probability that both $S_{i,1}^R$ and $S_{i,2}^R$ are $>$ individual I_i's contribution to the score of a random irrelevant SNP. This probability is

$$P(\min\{S_{i,1}^R, S_{i,2}^R\} > S_i^I) \quad = \quad \int_{-\infty}^{\infty} \phi_*^I(x)(1-\Phi_*^R(x))^2 dx \tag{6}$$

where ϕ_*^I is the PDF of S_i^I, so a gaussian with mean and variance as above, and Φ_*^R is the CDF of S_i^R. Machine computation shows that $P(\min\{S_{i,1}^R, S_{i,2}^R\} > S_i^I)$ is largest at b and decreases on $[b, N_I+2]$, very slowly near b.

An accurate and standard measure of the effectiveness of SURF, the success rate, is

$$\mathcal{P}(n) = P\Big(\min\Big\{\sum_{i=1}^N S_{i,1}^R, \sum_{i=1}^N S_{i,2}^R\Big\} > n\% \text{ of all SNP scores}\Big).$$

This is difficult to compute since the score of an irrelevant SNP is $\sum_{i=1}^N S_i^I$, and the S_i^I are not independent random variables. Nor are the S_i^R. Indeed, let $N_r(I_k, H)$ be the set of all hits within distance r of I_k, and $N_r(I_k, M)$ all such misses. Then if I_i and I_j are relatively close individuals, their neighborhoods $N_r(I_i, H)$ and $N_r(I_j, H)$ tend to be similar for large r, as do $N_r(I_i, M)$ and $N_r(I_j, M)$. Thus if the state of an irrelevant SNP agrees in individuals I_i and I_j, its scores S_i^I and S_j^I are somewhat correlated. This correlation decreases as r decreases. Thus the variances of $\sum S_i^R$ and $\sum S_i^I$ decrease more quickly with r than equations (2) and (3) might indicate. Since $P(\min\{S_{i,1}^R, S_{i,2}^R\} > S_i^I)$ is slowly decreasing near b, we thus expect that the optimal values of d are somewhat $> b$.

If ϕ^R and ϕ^I, the PDFs of $\sum_i S_i^R$ and $\sum_i S_i^I$, respectively, are known, then the success rate can be computed via

$$\mathcal{P}(n) = \int_{\ell(n)}^{\infty} \phi_{\min}^R(x)\,dx \tag{7}$$

where $\ell(n) = (\Phi^I)^{-1}(\frac{n}{100})$, $\Phi^I(t) = \int_{-\infty}^t \phi^I(x)\,dx$ being the CDF of $\sum_i S_i^I$, and $\phi_{\min}^R(x) = \frac{d}{dx}\Big(-\big(\int_x^\infty \phi^R(t)\,dt\big)^2\Big)$ is the PDF of $\min\Big\{\sum_{i=1}^N S_{i,1}^R, \sum_{i=1}^N S_{i,2}^R\Big\}$.

3 Relief using a single nearest neighbor

Next we analyze Relief. Let S be any of the sets $H_{j\Delta}$ or $M_{j\Delta}$ (or any subset of the set of individuals provided the states of the irrelevant SNPs of members of S are randomly assigned according to the given allele frequencies). Let $M(I_j, I_i)$, or just M_{ij}, denote the number of irrelevant SNPs of individuals I_i and I_j which match. We will find the probability density function of the random variable $\text{Max}_{|S|} = \max_{I_j} M(I_j, I_i)$, where I_j ranges over S and I_i is fixed. Note that if we disregard relevant SNPs, then $N_I - \text{Max}_{|S|}$ is the distance from I_i to its closest neighbor in S.

Set

$$\Phi(z) = \frac{1}{\sqrt{2\pi}} \int_{-\infty}^{b(z)} e^{-\frac{1}{2}x^2} dx, \quad \text{where} \quad b(z) = \frac{z + \frac{1}{2} - N_I ||\vec{P}||^2}{\sqrt{N_I ||\vec{P}||^2 (1 - ||\vec{P}||^2)}}. \tag{8}$$

Then, as with the probability $p(d)$ in equation (2) above, we have, for an arbitrary pair of individuals I_i and I_j, the good approximation

$$P(M(I_i, I_j) \leq z) = \Phi(z). \tag{9}$$

Since the states of irrelevant SNPs among different individuals are independent

$$P(\text{Max}_{|S|} \leq z) = \big(P(M_{ij} \leq z)\big)^{|S|} = \big(\Phi(z)\big)^{|S|}$$

and so

$$P(\text{Max}_{|S|} = z) = \frac{d}{dz}\Big(\big(P(M_{ij} \leq z)\big)^{|S|}\Big) = \frac{d}{dz}\Big(\big(\Phi(z)\big)^{|S|}\Big).$$

Next we define probabilities $P_{CH}(n\Delta)$ and $P_{CM}(n\Delta)$ for $n = 0$, 1 and 2. These are analogous to the $P_H(n\Delta)$ and $P_M(n\Delta)$ above, but use *closest* hits and misses rather than arbitrary ones. Specifically, $P_{CH}(n\Delta)$ is the probability that exactly n relevant SNPs change state in going from an arbitrary individual to a (random) closest neighbor in the same class. Closest misses are used for $P_{CM}(n\Delta)$. We express the $P_{CH}(n\Delta)$ and $P_{CM}(n\Delta)$ in a number of different forms. All rely on the fact that if $I_j \in M_{k\Delta}$, then the number of SNPs of I_i and I_j which match is $M_{ij} + (2 - k)$. Abbreviating $P(\text{Max}_{|M_{n\Delta}|} = z)$ to $PM_{|M_{n\Delta}|}(z)$ we have

$$\begin{aligned}
P_{CM}(2\Delta) &= \int_{z=0}^{\infty} \int_{y=0}^{z-1} \int_{x=0}^{z-2} PM_{|M_{2\Delta}|}(z)\, PM_{|M_{1\Delta}|}(y)\, PM_{|M_{0\Delta}|}(x) dx\, dy\, dz \\
&= \int_0^{\infty} \big(\Phi(z-2)\big)^{|M_{0\Delta}|} \big(\Phi(z-1)\big)^{|M_{1\Delta}|} \frac{d}{dz}\Big(\big(\Phi(z)\big)^{|M_{2\Delta}|}\Big) dz \\
&= \int_0^{\infty} P(\text{Max}_{|M_{0\Delta}|} \leq z-2) P(\text{Max}_{|M_{1\Delta}|} \leq z-1) P(\text{Max}_{|M_{2\Delta}|} = z) dz
\end{aligned}$$

In general,

$$P_{CM}(k\Delta) = \int_0^{\infty} \big(\Phi(z-2+i)\big)^{|M_{i\Delta}|} \big(\Phi(z-2+j)\big)^{|M_{j\Delta}|} \frac{d}{dz}\Big(\big(\Phi(z-2+k)\big)^{|M_{k\Delta}|}\Big) dz \tag{10}$$

where $k = 0, 1$ or 2, and $\{i, j, k\} = \{1, 2, 3\}$. Changing each M to H gives analogous formulas for the $P_{CH}(k\Delta)$. As a check, we note that

$$\begin{aligned}
1 &= \int_0^{\infty} \frac{d}{dx}\Big\{ \big(\Phi(x)\big)^{|M_{2\Delta}|} \big(\Phi(x-1)\big)^{|M_{1\Delta}|} \big(\Phi(x-2)\big)^{|M_{0\Delta}|} \Big\} dx \\
&= P_{CM}(2\Delta) \;+\; P_{CM}(1\Delta) \;+\; P_{CM}(0\Delta).
\end{aligned}$$

For the version of Relief in which only a single nearest neighbor is used, individual I_i's contribution to the score of a relevant SNP is given by the random variable $U_i^R = b(1, P_{CM}^R(\Delta)) - b(1, P_{CH}^R(\Delta))$. Here $P_{CM}^R(\Delta) = \frac{1}{2} P_{CM}(1\Delta) + P_{CM}(2\Delta)$, the probability that SNP 1 changes state in going from I_i to a (random) closest miss, and $P_{CH}^R(\Delta) = \frac{1}{2} P_{CH}(1\Delta) + P_{CH}(2\Delta)$. The mean of U_i^R is

$$M(U_i^R) = P_{CM}^R(\Delta) - P_{CH}^R(\Delta) = \sum_{i=0}^{2} \frac{i}{2}\big(P_{CM}(i\Delta) - P_{CH}(i\Delta)\big)$$

and the variance is

$$V(U_i^R) = P_{CM}^R(\Delta)(1 - P_{CM}^R(\Delta)) + P_{CH}^R(\Delta)(1 - P_{CH}^R(\Delta)).$$

For the score of an irrelevant SNP, say SNP k, we first define Φ_1 just as Φ was, but with $N_I - 1$ in place of N_I in equation (8). Let

$$F_M(x) = P_M(2\Delta)\Phi_1(x) + P_M(1\Delta)\Phi_1(x-1) + P_M(0\Delta)\Phi_1(x-2).$$

This is the probability that given a random individual $I_j \in M_\Delta$, the number of SNPs, both relevant and irrelevant, of I_i and I_j which agree is $\leq x$. Note that $F_M(x-1)$ is the same probability, but for $I_j \in M_\Sigma$, the set consisting of all misses with SNP k in the same state as that of individual I_i. The probability that SNP k of a random closest miss does not match SNP k of individual I_i is

$$P_{CM}(\Delta) = \int_{-\infty}^{\infty} \left\{ \frac{d}{dx}(F_M(x))^{|M_\Delta|} \right\} (F_M(x-1))^{|M_\Sigma|} dx.$$

From now on we omit those expressions involving hits which can be obtained from the analogous displayed ones by changing each M to H.

Individual I_i's contribution to the score of SNP k is $U_i^I = b(1, P_{CM}(\Delta)) - b(1, P_{CH}(\Delta))$ which has mean $P_{CM}(\Delta) - P_{CH}(\Delta)$ and variance $P_{CM}(\Delta)(1 - P_{CM}(\Delta)) + P_{CH}(\Delta)(1 - P_{CH}(\Delta))$.

4 ReliefF with n nearest neighbors

Relief algorithms typically use 10 nearest neighbors, rather than just one. To analize this, let $P_{CM}(c_1\Delta, \ldots, c_n\Delta)$ be the probability that a (random) miss closest to individual I_i is in the set $M_{c_1\Delta}$, a (random) second closest miss is in $M_{c_2\Delta}$, etc., up to a random n^{th} closest miss is in $M_{c_n\Delta}$. For $k = 0$, 1 and 2, let $n_k = \sum_{i=1}^{n-1} \delta(k, c_i)$, the number of the $c_1, \ldots, c_{n-1}$ equal to k. Then, for each $(c_1, \ldots, c_n) \in \{0,1,2\}^n$, we have

$$P_{CM}(c_1\Delta, \ldots, c_n\Delta) = \prod_{k=0}^{2} \binom{|M_{k\Delta}|}{n_k} n_k!$$
$$\times \int_0^\infty R_{c_1 \ldots c_{n-1}}(x) \frac{\prod_{i=0}^2 \big(\Phi(x-2+i)\big)^{|M_{i\Delta}|-n_i}}{\big(\Phi(x-2+c_n)\big)^{|M_{c_n\Delta}|-n_{c_n}}} \frac{d}{dx}\Big(\big(\Phi(x-2+c_n)\big)^{|M_{c_n\Delta}|-n_{c_n}}\Big)\, dx.$$

Here $R_{c_1 \ldots c_n}(x)$ is the probability that for a random n-tuple $(I_1, \ldots, I_n)$ of distinct individuals in $M_{c_1\Delta} \times \ldots \times M_{c_n\Delta}$, we have $M(I_1, I_i) \geq \ldots \geq M(I_n, I_i) \geq x$. It can be given inductively by

$$R_{c_1 \ldots c_n}(x) = \int_{t=x}^{\infty} R_{c_1 \ldots c_{n-1}}(t) \frac{d}{dt}\big(\Phi(t-2+c_n)\big)\, dt$$

where

$$R_{c_1}(x) = \int_{t=x}^{\infty} \frac{d}{dt}\big(\Phi(t-2+c_1)\big)\, dt = 1 - \Phi(x-2+c_1).$$

Again, as a check, we note that

$$0 \quad = \quad \prod_{k=0}^{2} \binom{|M_{k\Delta}|}{n_k} n_k! \int_{-\infty}^{\infty} \frac{d}{dx}\Big\{ R_{c_1 \ldots c_{n-1}}(x) \prod_{i=0}^{2} \big(\Phi(x-2+i)\big)^{|M_{i\Delta}|-n_i} \Big\} dx$$

$$= \quad -P_{CM}(c_1\Delta,\ldots,c_{n-1}\Delta)+\sum_{i=0}^{2}P_{CM}(c_1\Delta,\ldots,c_{n-1}\Delta,i\Delta).$$

Now for ReliefF using n nearest neighbors, let T_i^M be individual I_i's contribution due to misses to the score of a relevant SNP. Then we have

$$P(T_i^M=k)=\sum_{c_i\Delta}P\Big(\textstyle\sum_{j=1}^n b\big(1,\frac{c_j}{2}\big)=k\Big)\,P_{CM}(c_1\Delta,\ldots,c_n\Delta),$$

where here and below $\sum_{c_i\Delta}$ indicates the sum over all 3^n possibilities for $c_1\Delta,\ldots,c_n\Delta$. So the mean of T_i^M is

$$\begin{aligned} M(T_i^M) &= \sum_{k=0}^{n}k\,P(T_i^M=k)=\sum_{c_i\Delta}\sum_{k=0}^{n}k\,P\Big(\textstyle\sum_{j=1}^n b\big(1,\frac{c_j}{2}\big)=k\Big)\,P_{CM}(c_1\Delta,\ldots,c_n\Delta)\\ &= \sum_{c_i\Delta}\frac{\sum_{j=1}^n c_j}{2}P_{CM}(c_1\Delta,\ldots,c_n\Delta). \end{aligned}$$

The variance is $V(T_i^M)=M((T_i^M)^2)-(M(T_i^M))^2$, where

$$\begin{aligned} M((T_i^M)^2) &= \sum_{c_i\Delta}\sum_{k=0}^{n}k^2\,P\left(\textstyle\sum_{j=1}^n b\Big(1,\frac{c_j}{2}\Big)=k\right)\,P_{CM}(c_1\Delta,\ldots,c_n\Delta)\\ &= \sum_{c_i\Delta}\left(\sum_{j=1}^{n}\frac{c_j}{2}\left(1-\frac{c_j}{2}\right)+\left(\frac{\sum_{j=1}^n c_j}{2}\right)^2\right)P_{CM}(c_1\Delta,\ldots,c_n\Delta). \end{aligned}$$

Thus $T_i^R=T_i^M-T_i^H$, individual I_i's contribution to the score of a relevant SNP, has mean

$$M(T_i^R)=\sum_{c_i\Delta}\frac{\sum_{j=1}^n c_j}{2}\big(P_{CM}(c_1\Delta,\ldots,c_n\Delta)-P_{CH}(c_1\Delta,\ldots,c_n\Delta)\big) \tag{11}$$

and variance

$$V(T_i^R)=V(T_i^M)+V(T_i^H). \tag{12}$$

We remark that, as with SURF, the random variables T_i^R are not indepedant since, for instance, the relation "is the nearest neighbor of" among individuals tends to be symmetric.

The discussion of irrelevant SNP scores parallels that of the relevant ones. Using analogous notation with an I, for irrelevant, appended we have, for each $(c_1,\ldots,c_n)\in\{0,1\}^n$,

$$\begin{aligned} P^I_{CM}(c_1\Delta,\ldots,c_n\Delta) &= \prod_{k=0}^{1}\binom{|M_k|}{m_k}m_k!\\ &\times\int_0^\infty R^I_{c_1\ldots c_{n-1}}(x)\frac{\prod_{i=0}^{1}\big(F_M(x-1+i)\big)^{|M_i|-m_i}}{\big(F_M(x-1+c_n)\big)^{|M_{c_n}|-m_{c_n}}}\frac{d}{dx}\Big(\big(F_M(x-1+c_n)\big)^{|M_{c_n}|-m_{c_n}}\Big)\,dx \end{aligned}$$

where $M_0=M_\Sigma$, $M_1=M_\Delta$, $m_k=\sum_{i=1}^{n-1}\delta(k,c_i)$, $R^I_{c_1\ldots c_n}(x)=\int_{t=x}^\infty R^I_{c_1\ldots c_{n-1}}(t)\frac{d}{dt}\big(F_M(t-1+c_n)\big)\,dt$ and $R^I_{c_1}(x)=\int_{t=x}^\infty\frac{d}{dt}\big(F_M(t-1+c_1)\big)\,dt=1-F_M(x-1+c_1)$.

So the mean and variance of T_i^I, individual I_i's contribution to an irrelevant SNP score, are

$$M(T_i^I)=\sum_{c_i\Delta}(\textstyle\sum_{i=1}^n c_i)\big(P^I_{CM}(c_1\Delta,\ldots,c_n\Delta)-P^I_{CH}(c_1\Delta,\ldots,c_n\Delta)\big)$$

and

$$V(T_i^I) = V(T_i^{M,I}) + V(T_i^{H,I})$$

where

$$V(T_i^{M,I}) = \sum_{c_i\Delta} \left(\sum_{i=1}^n c_i\right)^2 P_{CM}^I(c_i\Delta, \ldots, c_n\Delta) - \left(\sum_{c_i\Delta} \left(\sum_{i=1}^n c_i\right) P_{CM}^I(c_i\Delta, \ldots, c_n\Delta)\right)^2.$$

5 Examples

We conclude by using the ideas developed in this appendix to compare SURF with ReliefF using 10 nearest neighbors. We assume a sample size of 1600 and use the .1 heritability penetrance functions of models 19 and 17.

For relevant SNPs, equation (11) gives, for model 19, expected Relief scores of .01187, .02260 and .03068 using 1, 2 and 3 nearest neighbors, respectively. According to equation (12), variances of these scores are .49206, .98226 and 1.47110, respectively. For model 17, means are .00545, .01038 and .01503, and variances .49174, .98163 and 1.47019, respectively. Extrapolating to 10 nearest neighbors, we set $M(T_i^R) = .087$ and $V(T_i^R) = 4.865$ for model 19, and $M(T_i^R) = .046$ and $V(T_i^R) = 4.86$ for model 17. Means of irrelevant SNP scores are ≈ 0, and variances of relevant and irrelevant SNP scores are nearly the same. So we set $M(T_i^I) = 0$ and $V(T_i^R) = V(T_i^I)$ for both models.

For SURF we use the quantities given by equations (3) and (4) and the approximations of setting $M(S_i^I) = 0$ and $V(S_i^R) = V(S_i^I)$. We also set $d = 384$ which maximizes $M(S_i^R)$ as discussed just after equation (3).

We would now like to use equation (7), and its analog for ReliefF, to compute the success rates of the two methods, but don't know the PDFs ϕ^I and ϕ^R implicit in this equation. So we approximate these by assuming that the random variables S_i^I, as well as the S_i^R, are independent and use the Central Limit theorem. The PDFs of $\sum_i T_i^I$ and $\sum_i T_i^R$ are approximated similarly. Then equation (7) overestimates slightly the success rates since, as mentioned, the S_i^I and the S_i^R, as well as the T_i^I and T_i^R can be somewhat correlated. Machine computation using equation (7) and the approximate PDFs gives the success rates shown in Figure 1.

The quantity given by equation (1) for model 17 is .02502, which is about average for the five penetrance functions with heritability .1 used in our simulations. So the figure involving model 17 is representative of the success rates of the two methods. For model 19, equation (1) gives .05446, the highest value of the five. Figure 2 for model 19 is included to show how much success rates depend on penetrance functions, even those having the same heritability.

The number of nearest neighbors used by SURF in our simulations is, on the average, one fourth the sample size. Since SURF outperforms Relief, one wonders if Relief algorithms could be improved by using many more than the usual 10 nearest neighbors for detecting epistatic pairs. Our preliminary work here looks very promising.

Figure 1: Success rates computed using equation (7) for Model 17

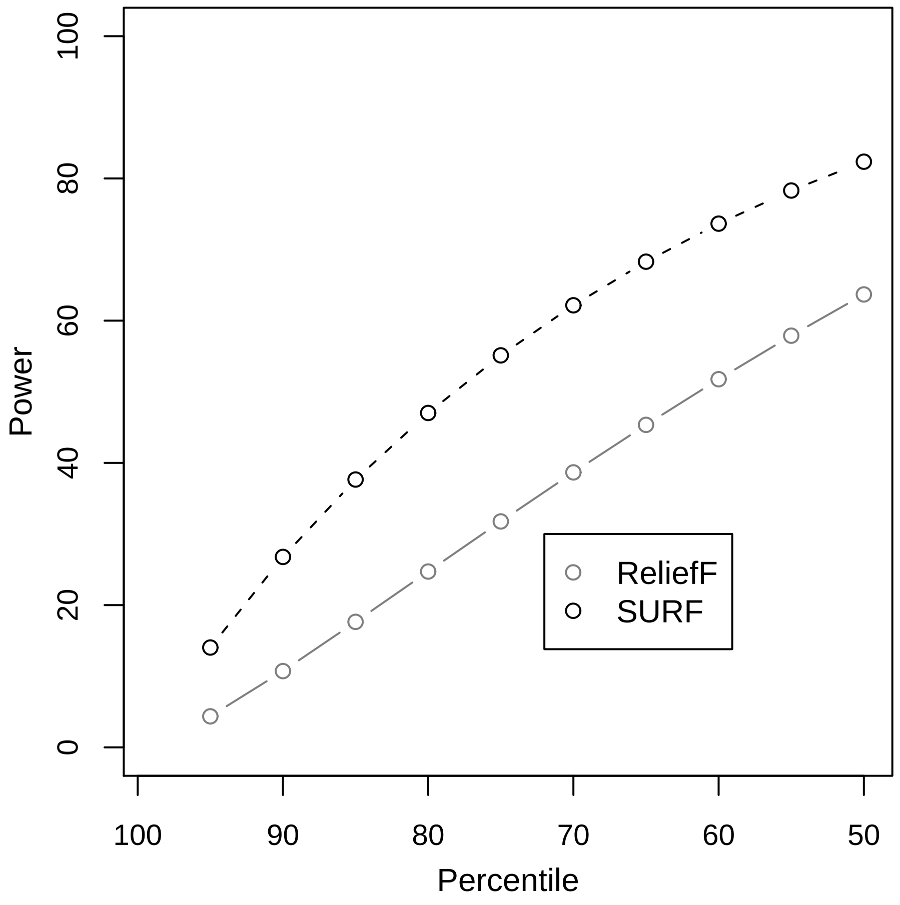

Figure 2: Success rates computed using equation (7) for Model 19

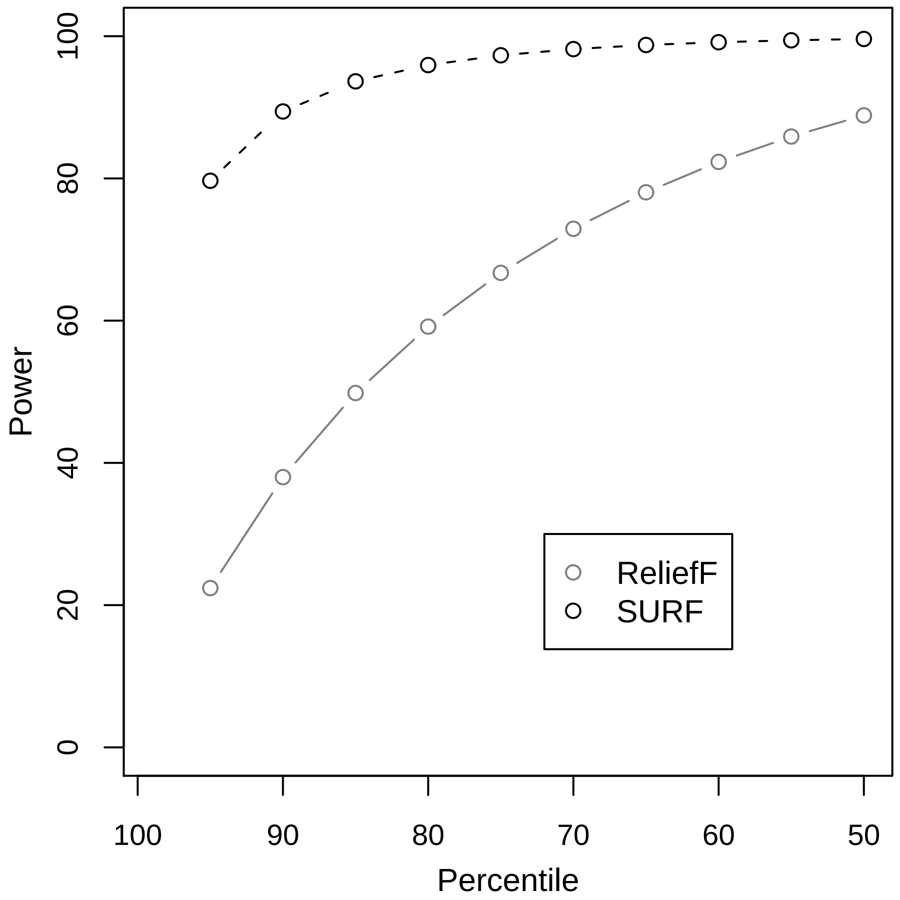

Supplement: Additional file 1 — Appendix. This is an appendix to accompany the manuscript that includes additional theoretical analysis of the Relief algorithms discussed in the manuscript. [file 1756-0381-2-5-S1.pdf]
