# Supplementary material for: Spatially Uniform ReliefF (SURF) for computationally-efficient filtering of gene-gene interactions
Source: BioData Min. 2009 Sep 22;2:5. doi: 10.1186/1756-0381-2-5 (PMC2761303; doi:10.1186/1756-0381-2-5)
Supplement: Additional file 2 — Epistasis models. These are the epistasis models used in our data simulation. [file 1756-0381-2-5-S2.pdf]

Model: 1
Minor Allele Frequency: 0.4
Heritability: 0.4000
0.07740046965062726 0.65629148224594369 0.88043034227588512
0.89171791076660167 0.23502330107456088 0.31202064327909118
0.17415105671391135 0.84173010745057153 0.10642564576961248

Model: 2
Minor Allele Frequency: 0.4
Heritability: 0.4000
0.89463325168775487 0.32250355983125994 0.16083294412364132
0.067851398302161095 0.72758757664961526 0.80584006378616135
0.92478942871093761 0.23287270034568899 0.36187412427819299

Model: 3
Minor Allele Frequency: 0.4
Heritability: 0.4000
0.80496340381855858 0.25066842838209502 0.084732989875637751
0.0021183247468909553 0.66827261529001236 0.63832185706313771
0.83038330078124989 0.079084123883928534 0.54229113520408156

Model: 4
Minor Allele Frequency: 0.4
Heritability: 0.4000
0.30747244009387348 0.68195810431077042 0.95789490952322121
0.99731445312499978 0.39025348165760865 0.2808642481626728
0.011825863080533594 0.99041603299468861 0.69772592175148196

Model: 5
Minor Allele Frequency: 0.4
Heritability: 0.4000
0.083441185930554801 0.89081192016601562 0.037084971524691028
0.61885546231828159 0.27137700516766089 0.6907075946473703
0.85295434506789358 0.07880556448996838 0.74169943049382059

Model: 6
Minor Allele Frequency: 0.4
Heritability: 0.3000
0.89071429217303244 0.36161477673691483 0.47961538809317128
0.2131623947080761 0.82917633893885567 0.60142247078350042
0.92497253417968761 0.26740461121861325 0.68516484013310186

Model: 7
Minor Allele Frequency: 0.4
Heritability: 0.3000
0.076503403007486065 0.68923899191003646 0.41651852748520191
0.762908935546875 0.14964207301232804 0.49089683596470229
0.19550869657468664 0.65665420914758887 0.246510965246344

Model: 8
Minor Allele Frequency: 0.4
Heritability: 0.3000
0.13216475238950232 0.79298851433701389 0.27376984423539763

0.79928207397460926 0.21266986942040947 0.5137118054187203
0.25488916532261163 0.52787231460330974 0.79298851433701378

Model: 9
Minor Allele Frequency: 0.4
Heritability: 0.3000
0.61050997075346336 0.10436067876127574 0.75922393798828136
0.18002217086320069 0.6739960503332395 0.018915373025481283
0.53223946168250658 0.18915373025481233 0.68095342891732447

Model: 10
Minor Allele Frequency: 0.4
Heritability: 0.3000
0.09085222630718956 0.82713381033837152 0.86309614991830086
0.86877441406250022 0.39321979198580487 0.41451328252655228
0.7381743387459152 0.50820464090584161 0.36340890522875824

Model: 11
Minor Allele Frequency: 0.4
Heritability: 0.2000
0.35629969692686531 0.89074924231716313 0.80852623533404044
0.95470046997070312 0.50780320053465633 0.61096262133292611
0.61667255237342067 0.75485288355339075 0.63037638687060782

Model: 12
Minor Allele Frequency: 0.4
Heritability: 0.2000
0.086034334622896627 0.53641104086851465 0.64134685809795666
0.67719449752416372 0.27466895845201272 0.096462738819611371
0.21899648813100964 0.41322551629482157 0.71173858642578125

Model: 13
Minor Allele Frequency: 0.4
Heritability: 0.2000
0.85545713577799776 0.33861844957879078 0.77228769202180358
0.51287823649653119 0.65066888240213083 0.60693891693508417
0.24950833126858268 0.99902343749999989 0.15445753840436072

Model: 14
Minor Allele Frequency: 0.4
Heritability: 0.2000
0.50572337478887852 0.83797187101645587 0.023522017432040864
0.60275169669604722 0.45418728798464991 0.95656204223632846
0.72918254039326669 0.42731665001540908 0.75270455782530765

Model: 15
Minor Allele Frequency: 0.4
Heritability: 0.2000
0.39325632337707184 0.76398233656066561 0.66362004569880872
0.85000715729940013 0.39820616425291167 0.73325918629683184
0.40554558348260533 0.92681503295898438 0.14747112126640194

Model: 16

Minor Allele Frequency: 0.4
Heritability: 0.1000
0.13704285191875668 0.48380279541015619 0.18687661625285001
0.48172638856290229 0.16576647997243546 0.3654476051166845
0.19310583679461168 0.36077568971036328 0.42981621738155501

Model: 17
Minor Allele Frequency: 0.4
Heritability: 0.1000
0.46944863455636154 0.19816889081682473 0.75419616699218739
0.33669471740722651 0.50210267778426876 0.14109111967540922
0.33861868722098215 0.45277422950381324 0.28474753243582585

Model: 18
Minor Allele Frequency: 0.4
Heritability: 0.1000
0.4777033379737366 0.31081338977137335 0.86354064941406228
0.38736841188254917 0.57862837545696999 0.26334927606244457
0.63387558308053504 0.43559807581259002 0.13779903980011632

Model: 19
Minor Allele Frequency: 0.4
Heritability: 0.1000
0.06836809430803574 0.29911041259765636 0.017092023577008935
0.28885519845145102 0.044391783456953736 0.28515192667643241
0.047857666015625022 0.26207769484747034 0.17433864048549114

Model: 20
Minor Allele Frequency: 0.4
Heritability: 0.1000
0.539337158203125 0.12017838851265285 0.25794385827105976
0.16512315169624661 0.37836651180101483 0.32536100304645038
0.12310956872027853 0.42648672020953632 0.27553093951681384

Model: 21
Minor Allele Frequency: 0.4
Heritability: 0.0500
0.0021849651725924743 0.15458628596091756 0.21412658691406247
0.19901391113696451 0.071056888217017763 0.021849651725924731
0.080843711385921543 0.12181180837203044 0.13546784070073339

Model: 22
Minor Allele Frequency: 0.4
Heritability: 0.0500
0.18817138671875003 0.019514069733796293 0.1714450412326389
0.032058828848379621 0.1742327654803241 0.058542209201388903
0.13381076388888891 0.086767917209201392 0.091994900173611113

Model: 23
Minor Allele Frequency: 0.4
Heritability: 0.0500
0.0051233719806281896 0.17931801932198663 0.25104522705078131
0.21091214653586049 0.099834595678074317 0.026470755233245637
0.15626284540915977 0.097557541464461758 0.15626284540915977

Model: 24
Minor Allele Frequency: 0.4
Heritability: 0.0500
0.17351901146673387 0.32052817395938343 0.15423912130376347
0.22252206563095037 0.25351716683044806 0.24501527082108257
0.44825744628906256 0.024501527082108236 0.4241575835853495

Model: 25
Minor Allele Frequency: 0.4
Heritability: 0.0500
0.098139299665178537 0.21892613002232134 0.30196707589285704
0.30196707589285698 0.12623901367187493 0.12141592843191962
0.052844238281249983 0.30825805664062494 0.13588518415178566

Model: 60
Minor Allele Frequency: 0.4
Heritability: 0.0250
0.16602186723188922 0.16548974586255621 0.12770912863991477
0.11440609440659028 0.19905773557797829 0.14314064835057111
0.2809600830078125 0.028202432574647839 0.2809600830078125

Model: 26
Minor Allele Frequency: 0.4
Heritability: 0.0250
0.10836029052734375 0.0061112285061717415 0.079902032409051457
0.025813099511143335 0.079423167488791704 0.045697395247642443
0.020796419394136678 0.090026605008828517 0.025174612950797034

Model: 27
Minor Allele Frequency: 0.4
Heritability: 0.0250
0.0058008567574097929 0.093780517578125 0.0077344756765463912
0.0789561058647444 0.016194058447769003 0.075894542576111459
0.052207710816688138 0.04253961622100514 0.057041758114529627

Model: 28
Minor Allele Frequency: 0.4
Heritability: 0.0250
0.19945032456341907 0.071580505371093728 0.16753827263327203
0.085763639562270191 0.1873540317311006 0.076012734805836382
0.12498887005974262 0.10792478673598341 0.22604370117187494

Model: 29
Minor Allele Frequency: 0.4
Heritability: 0.0250
0.16478257768609553 0.095632745978537562 0.2618865966796875
0.16625385070114995 0.15133677707629259 0.091464139102550024
0.050023282511850425 0.25036162472842788 0.055908374572068126
