# Supplementary figures and images for: Spatially Uniform ReliefF (SURF) for computationally-efficient filtering of gene-gene interactions
Source: BioData Min. 2009 Sep 22;2:5. doi: 10.1186/1756-0381-2-5 (PMC2761303; doi:10.1186/1756-0381-2-5)

**Population Size: 800**

## 99th percentile

## 90th percentile

### 75th percentile

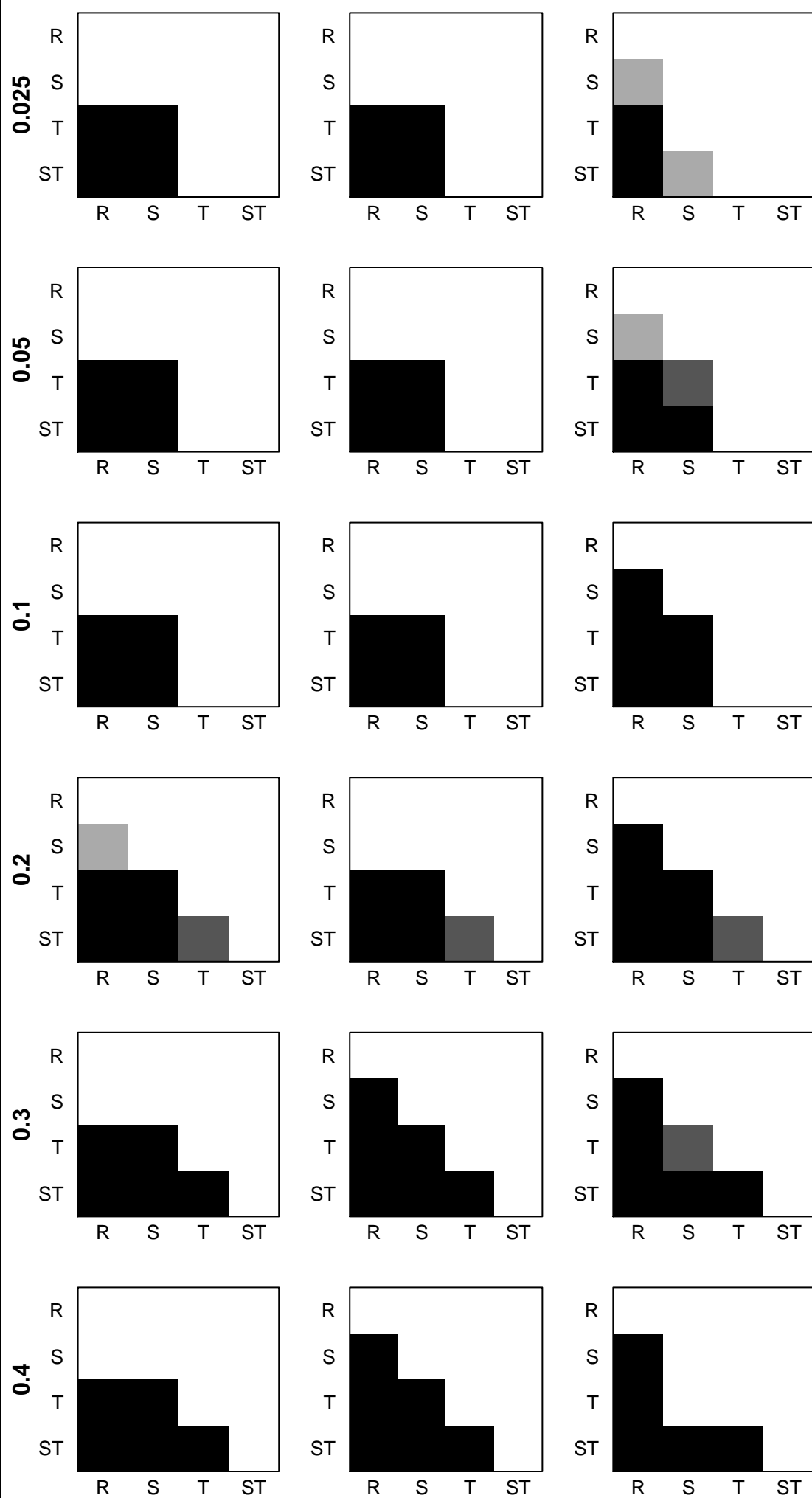

Supplement: Additional file 3 — Significance of differences with a sample size of 800. This is a plot showing the significance of statistical results for the situation where there are 400 cases and 400 control individuals. These plots follow the example shown in Figure 2. Pairwise comparisons are made between each pair of methods at the 99th, 95th, and 75th percentiles. ReliefF, SURF, TuRF, and SURF & TuRF are labeled R, S, T, and ST respectively. Significance is illustrated with levels of grey (i.e. light grey indicates 0.01 <p ≤ 0.05, dark grey indicates 0.001 <p ≤ 0.01, and black indicates p ≤ 0.001). [file 1756-0381-2-5-S3.pdf]

**Population Size: 1600**

### 99th percentile

**90th percentile**

**75th percentile**

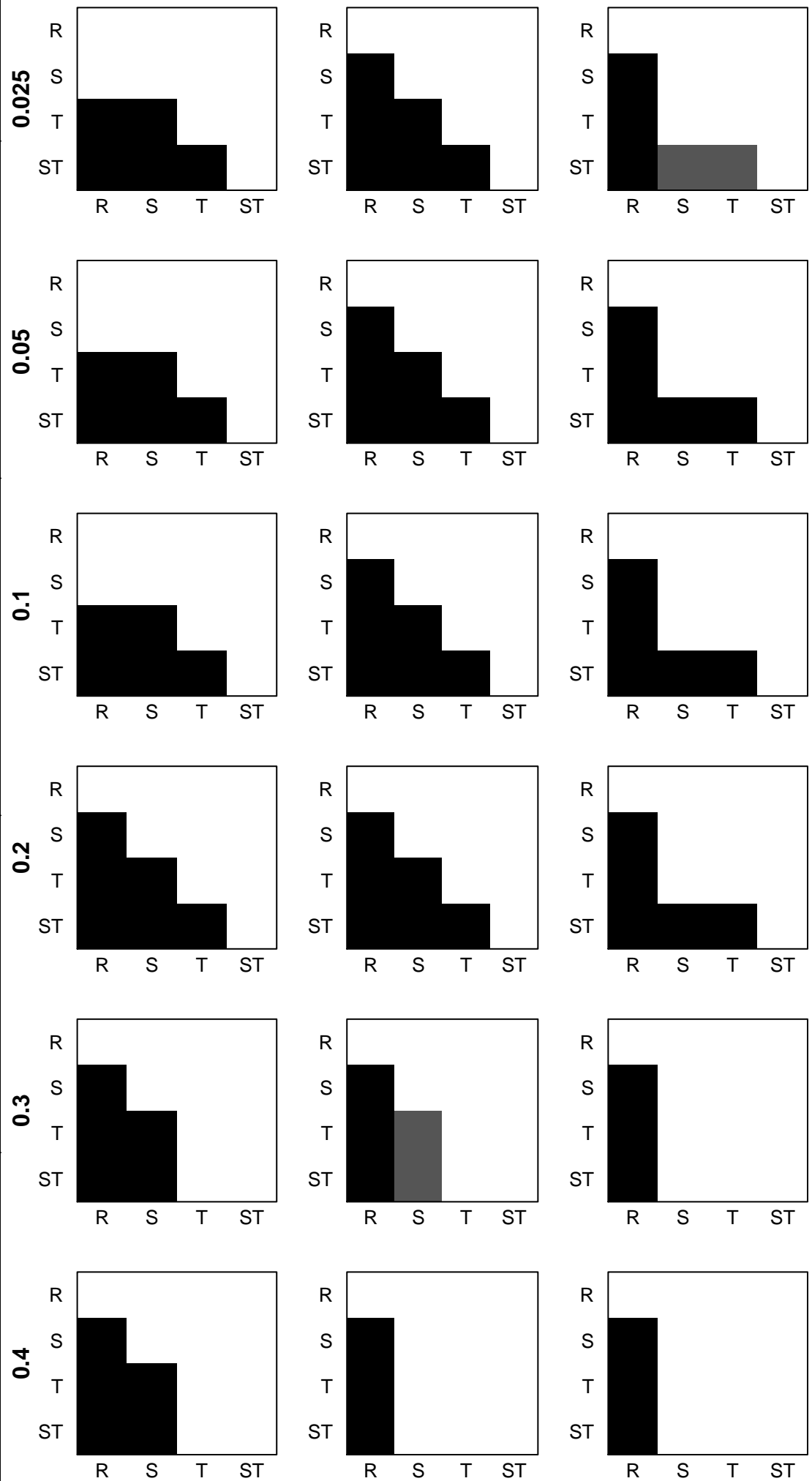

Supplement: Additional file 4 — Significance of differences with a sample size of 1600. This is a plot showing the significance of statistical results for the situation where there are 800 cases and 800 control individuals. These plots follow the example shown in Figure 2. Pairwise comparisons are made between each pair of methods at the 99th, 95th, and 75th percentiles. ReliefF, SURF, TuRF, and SURF&TuRF are labeled R, S, T, and ST respectively. Significance is illustrated with levels of grey (i.e. light grey indicates 0.01 <p ≤ 0.05, dark grey indicates 0.001 <p ≤ 0.01, and black indicates p ≤ 0.001). [file 1756-0381-2-5-S4.pdf]

**Population Size: 3200**

### 99th percentile

### 90th percentile

**75th percentile**

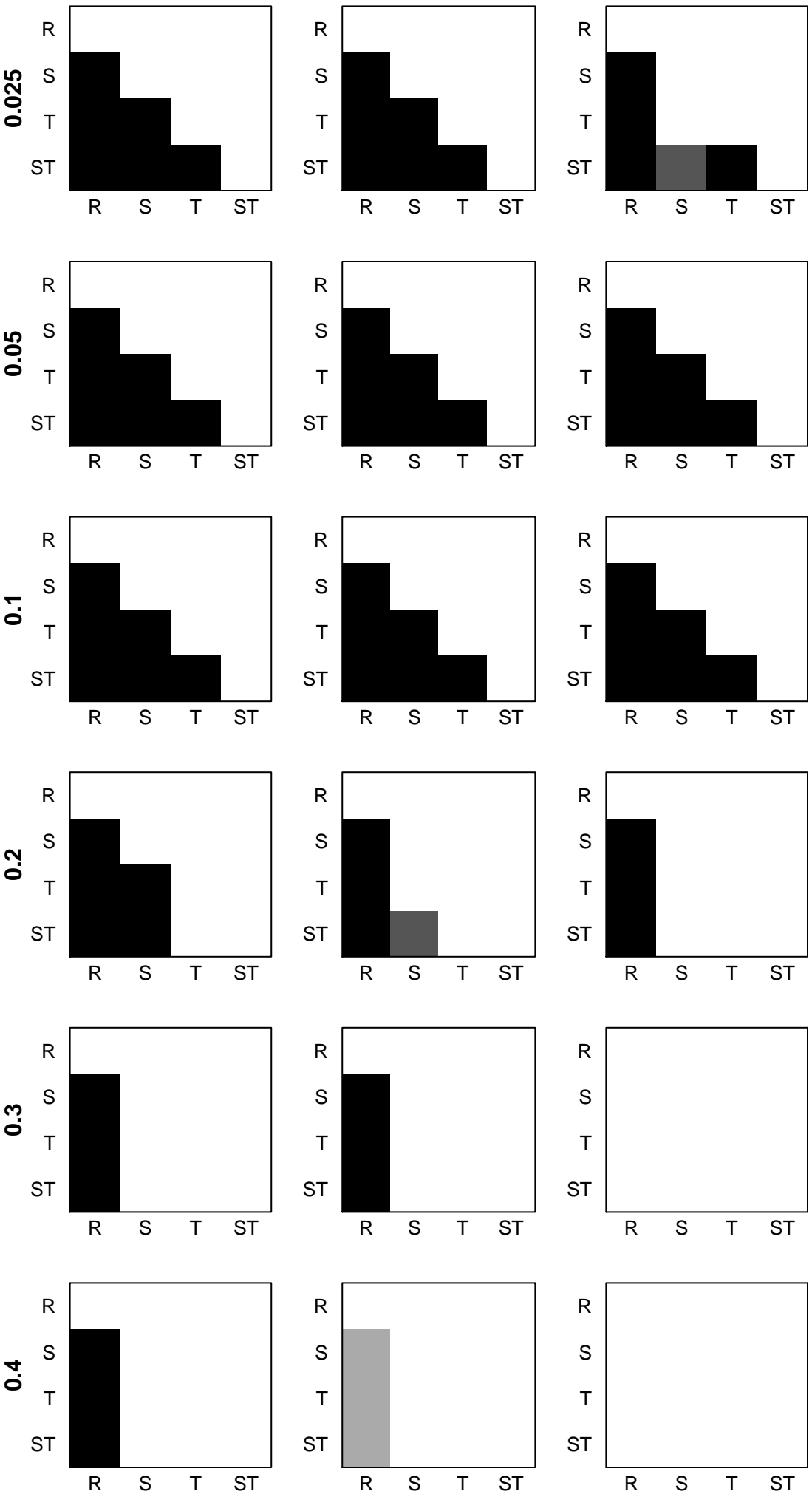

Supplement: Additional file 5 — Significance of differences with a sample size of 3200. This is a plot showing the significance of statistical results for the situation where there are 1600 cases and 1600 control individuals. These plots follow the example shown in Figure 2. Pairwise comparisons are made between each pair of methods at the 99th, 95th, and 75th percentiles. ReliefF, SURF, TuRF, and SURF&TuRF are labeled R, S, T, and ST respectively. Significance is illustrated with levels of grey (i.e. light grey indicates 0.01 <p ≤ 0.05, dark grey indicates 0.001 <p ≤ 0.01, and black indicates p ≤ 0.001). [file 1756-0381-2-5-S5.pdf]
